# Training health care professionals in root cause analysis: a cross-sectional study of post-training experiences, benefits and attitudes

**DOI:** 10.1186/1472-6963-13-50

**Published:** 2013-02-07

**Authors:** Paul Bowie, Joe Skinner, Carl de Wet

**Affiliations:** 1 Postgraduate General Practice Education, NHS Education for Scotland, 2 Central Quay, Glasgow, Scotland, G3 8BW, United Kingdom; 2 NHS Territorial Board (Anonymised), Scotland, United Kingdom

## Abstract

**Background:**

Root cause analysis (RCA) originated in the manufacturing engineering sector but has been adapted for routine use in healthcare to investigate patient safety incidents and facilitate organizational learning. Despite the limitations of the RCA evidence base, healthcare authorities and decision makers in NHS Scotland – similar to those internationally - have invested heavily in developing training programmes to build local capacity and capability, and this is a cornerstone of many organizational policies for investigating safety-critical issues. However, to our knowledge there has been no systematic attempt to follow-up and evaluate post-training experiences of RCA-trained staff in Scotland. Given the significant investment in people, time and funding we aimed to capture and learn from the reported experiences, benefits and attitudes of RCA-trained staff and the perceived impact on healthcare systems and safety.

**Methods:**

We adapted a questionnaire used in a published Australian research study to undertake a cross sectional online survey of health care professionals (e.g. nursing & midwifery, medical doctors and pharmacists) formally trained in RCA by a single territorial health board region in NHS Scotland.

**Results:**

A total of 228/469 of invited staff completed the survey (48%). A majority of respondents had yet to participate in a post-training RCA investigation (n=127, 55.7%). Of RCA-experience staff, 71 had assumed a lead investigator role (70.3%) on one or more occasions. A clear majority indicated that their improvement recommendations were generally or partly implemented (82%). The top three barriers to RCA success were cited as: lack of time (54.6%), unwilling colleagues (34%) and inter-professional differences (31%). Differences in agreement levels between RCA-experienced and inexperienced respondents were noted on whether a follow-up session would be beneficial after conducting RCA (65.3% v 39.4%) and if peer feedback on RCA reports would be of educational value (83.2% v 37.0%). Comparisons with the previous research highlighted significant differences such as less reported difficulties within RCA teams (P<0.001) and a greater proportion of respondents taking on RCA leadership roles in this study (P<0.001).

**Conclusion:**

This study adds to our knowledge and understanding of the need to improve the effectiveness of RCA training and frontline practices in healthcare settings. The overall evidence points to a potential organisational learning need to provide RCA-trained staff with continuous development opportunities and performance feedback. Healthcare authorities may wish to look more critically at whom they train in RCA, and how this is delivered and supported educationally to maximize cost-benefits, organizational learning and safer patient care.

## Background

Root cause analysis (RCA) is a structured approach to the investigation of patient safety incidents that is commonly applied in many modern health systems worldwide, particularly in acute hospital settings [1]. The RCA technique originated in the engineering industry as a method of identifying latent systems-based issues that contributed to underperformance, variations or design failures in mechanical production processes [2]. Its inherent principles have been adapted in many high reliability organisations – such as the petro-chemical, nuclear power, aerospace and aviation industries – to systematically uncover and improve underlying systems problems, and ergonomic and cultural issues identified as contributory factors in work-related accidents and incidents [3,4].

In healthcare, safety-based RCA investigations (or variants of this approach) were first introduced in the 1990s to facilitate organisational learning [5]. There is general consensus that RCA utilises a ‘*toolbox rather than a single method*’ with team-led investigations typically attempting to ascertain the ‘what, how and why’ of identified patient safety incidents [4]. To achieve this, a small multi-disciplinary team of appointed investigators often draws on a multitude of analytical and problem-solving techniques [4-6] such as Brainstorming, Pareto Analysis, the Five-Whys technique and Fault-Tree-Analysis to accomplish these aims using recommended step-wise processes (Table 1). Once ‘root causes’ are established by investigators, different levels of local team-based and wider organizational learning needs are determined and a series of improvement recommendations formulated which, if implemented, should minimize the risk of incident recurrence [2-5]. It should be noted that, broadly speaking, in general medical practice settings particularly in the United Kingdom (UK), significant event analysis (a less rigorous investigative method based on reflective learning theory) rather than RCA is the routinely applied technique of choice for historical and feasibility reasons [7].

**Table 1:** The seven steps for root cause analysis (RCA) team investigation details

	**Seven Steps to RCA [**[2]**]**	**Team-based RCA Incident Investigations [**[6,][8]**]**
**1.**	Identify the incident to be analysed	Small operational team appointed
**2.**	Organise a team to carry out the RCA	Agree terms of reference
**3.**	Study the work processes	Agree methods for gathering evidence
**4.**	Collect the facts	Interrogate, discuss and analyse evidence
**5.**	Search for causes	Draft recommendations for service improvement
**6.**	Take action	
**7.**	Evaluate the actions taken	

The evidence base underpinning the effectiveness of RCA in healthcare as a method to gain an in-depth understanding of safety issues and facilitate improvements to prevent future incidents is equivocal [9,10]. Controlled trials to test the efficacy of the RCA framework are lacking. Some individual studies of single incident investigations report positive evidence such as the implementation of ‘strong’ corrective actions to prevent recurrence of events as judged by the authors of a recent RCA review in medicine [9].

However in an evaluation of 445 RCAs undertaken in New South Wales to identify and theme learning needs related to patient, human (staff) and systems factors, the authors concluded that the effectiveness of RCA as a means by which staff can achieve the desired improvements in patient care that were recommended was limited [10]. A recent literature review of RCA effectiveness by Percarpio et al. (2008) identified a small number of formal published studies of relevance [11]. They highlighted ‘*numerous theoretical problems with the analytical framework*’ and called for more research ‘*at the system level and cost-benefits analysis…to determine the effectiveness of RCA*’. Additionally, Vincent (2004) describes the RCA methodological approach as ‘misleading’ and suggests that “*incident analysis, properly understood, is not a retrospective search for root causes*” but should be framed in terms of the incident acting as a ‘*window*’ on the ‘*gaps and inadequacies*’ of the healthcare system [6].

Despite this, it is evident that healthcare authorities and decision-makers have high expectations for the transferability of RCA as an improvement tool, particularly since it seems to be successfully established in non-healthcare industry to investigate safety-critical issues, albeit in arguably more linear and less complex institutional settings [12-15]. Moreover a range of external healthcare bodies with regulatory, accreditation or quality management oversights expect care provider organisations to have transparent incident reporting and investigation mechanisms in place. In the past decade the National Patient Safety Agency in England and Wales has strongly promoted the adoption of RCA and developed an in-depth training programme and online educational resources to build capacity in this area and support healthcare organisations and staff in explicit efforts to make patient care safer [16,17].

Similarly in the National Health Service in Scotland (NHSiS) many territorial health authorities have invested heavily in providing internal or external training in RCA methods to a range of staff groups, and this is a cornerstone of their organizational policies for investigating patient safety incidents. However, to our knowledge there has been no systematic attempt to follow-up and evaluate the post-training experiences, benefits and attitudes of NHSiS staff who have received formal instruction in RCA methods, and then put this knowledge into practice in the workplace when required to by their employing organisation. Given this significant investment in people, time and funding, it is important to capture and learn from the reported outcomes of this type of educational intervention, and its subsequent impact in the healthcare system and on improving patient safety.

In this study we aimed to:

1. Measure the extent to which respondents had subsequently participated in formal RCA investigations and also assumed a lead investigator role.

2. Determine the extent to which improvement recommendations arising from RCA investigations were actually implemented.

3. Ascertain if respondents encountered a range of barriers to RCA practice previously cited in the literature.

4. Establish participants’ perceptions of the adequacy of RCA training provided and their attitudes to the value of the tool as an improvement method in the healthcare workplace.

5. Compare relevant aspects of our findings with those reported in a previous Australian study [18] as one way of gauging progress with RCA training and impact in a different setting and at a different point in time.

## Methods

### Design, participants and setting

Cross sectional design utilizing an online questionnaire survey of healthcare professionals (e.g. medical and nursing & midwifery) based in a single (anonymised) territorial health board region in NHSiS who had attended RCA training either internally-run or externally sanctioned by the clinical risk department in a previous 36-month period. We left a 2-month gap to allow newly-trained staff the opportunity to experience involvement in RCA investigations.

### Data collection

We adapted a questionnaire used in the aforementioned Australian study [18] by Braithwaite et al. (2006) and piloted this with six colleagues who had previously attended RCA training. Minor alterations to questionnaire wording and style were then made to suit local circumstances. For example, we altered the rating scale for two items: ‘*RCAs should be conducted by colleagues with a clinical background and not by staff out-with your department*’ and ‘*patients and relatives should be part of the RCA team*’, to free-text responses to provide opportunities for more detailed answers from respondents. We added a statement ‘*when you were involved in an RCA(s), to what extent did you encounter interference from internal/external sources’*, to reflect a recent research finding. Unlike the Australian survey we included those healthcare professionals who were trained in RCA but did not subsequently participate in or lead an incident investigation and compared their responses with those who had.

We identified staff names and email addresses from the organizational database of all those who had attended RCA training in the chosen study period. During May and June 2011, we emailed a cover note explaining the study purpose and the online link to the web-based survey tool (QuestBack) to all participants. Non-respondents were followed up on three occasions via automatically generated email reminders.

Data were collected from respondents on: participation rates and leadership roles in RCA investigations; the extent to which RCA improvement recommendations were implemented; encountered barriers to conducting RCA; and attitudes to the adequacy of RCA training and the analytical process. A range of Likert-type scales was used to assess attitudinal strength. Free text responses were thematically analysed [19] by the authors independently with consensus reached over any discrepancies.

### Statistical analysis

The data were coded in Microsoft Excel software and exported to SPSS version 17.0. Characteristics of respondents including gender, professional groups, healthcare sector, job experience and RCA training details were summarized using simple descriptive statistics. We divided respondents into two groups: Group One consisted of those who had led or participated in one or more RCA investigations since training; and Group Two consisted of those who had not achieved either task. We compared group responses to attitudinal statements using chi-square analyses to determine statistical differences, and also Fisher’s Exact Test where necessary. Levene’s test was used to confirm the assumption of equal variance between groups. Differences in perceptions and characteristics between groups were considered statistically significant if p<0.05. We also calculated differences in proportions of responses to selected questionnaire items along with 95% confidence intervals and made direct comparisons with the findings of Braithwaite et al. (2006) [18].

### Ethical review

The study was pre-screened by the west of Scotland Research Ethics Committee but did not require formal ethical approval.

## Results

### Response rate, respondent characteristics and demographics

A total of 228/469 of invited healthcare professionals completed the survey (48%). Table 2 outlines details of respondent characteristics and demographics together with background information on when they attended RCA training, the type of training received and how long the training lasted. The great majority of participants were female (176, 77.2%), with the largest group of respondents coming from the nursing & midwifery professions (99, 43.4%), and those based in the acute sector (91, 39.9%). Most participants attended a one-day in-house RCA training event (181, 81.1%) .

**Table 2:** Respondent characteristics, demographics and RCA training details

**Characteristic**	**Participated or led RCA**		**Total**	**Chi-square**		
	**Yes n=101 (%)**	**No n=127 (%)**	**n=228 (%)**	**value**	**df**	**p-value**
**Training**						
**When did you attend RCA training?**				13.5	3	0.004
≤6 months ago	4 (4.0)	12 (9.4)	16 (7.0)			
7-11 months ago	8 (7.9)	25 (19.7)	33 (14.5)			
12-24 months ago	39 (38.6)	52 (40.9)	91 (39.9)			
>24 months ago	50 (49.5)	38 (29.9)	88 (38.6)			
**What type of training did you receive?**				0.486	2	0.784
NHS (in-house)	80 (79.2)	105 (82.7)	185 (81.1)			
Tutor	14 (13.9)	14 (11.0)	28 (12.3)			
Combination (e-learning, above, external)	7 (6.9)	8 (6.3)	15 (6.6)			
**How long was the training?**				1.156	2	0.561
≤One day	88 (87.1)	115 (90.6)	203 (89.0)			
1-2 days	12 (11.9)	10 (7.9)	22 (9.6)			
>2 days	1 (1.0)	2 (1.6)	3 (1.3)			
**Demographics**						
**Gender**				1.097	1	0.295
Male	25 (24.8)	24 (18.9)	49 (21.5)			
Female	75 (74.3)	101 (79.5)	176 (77.2)			
**Professional group**				10.082	4	0.039
Nursing	45 (44.6)	54 (42.5)	99 (43.4)			
Medical	8 (7.9)	6 (4.7)	14 (6.1)			
Management	30 (29.7)	24 (18.9)	54 (23.7)			
Pharmacy	7 (6.9)	20 (15.7)	27 (11.8)			
Other	10 (9.9)	23 (18.1)	33 (14.5)			
**Healthcare setting**				22.046	4	<0.001
Primary care	19 (18.8)	40 (31.5)	59 (25.9)			
Acute sector	55 (54.5)	36 (28.3)	91 (39.9)			
Mental health	13 (12.9)	22 (17.3)	35 (15.4)			
NHS Board HQ	8 (7.9)	6 (4.7)	14 (6.1)			
Other	5 (5.0)	22 (17.3)	27 (11.8)			
**Years experience in current job**				5.181	3	0.159
≤5 years	25 (24.8)	25 (19.7)	50 (21.9)			
6-10 years	22 (21.8)	17 (13.4)	39 (17.1)			
11-15 years	13 (12.9)	16 (12.6)	29 (12.7)			
>15 years	41 (40.6)	69 (54.3)	110 (48.2)			

We compared the professional characteristics and demographic details of Group One and Group Two respondents (Table 2). Three factors were statistically associated with a greater likelihood of involvement in or leading an RCA: being based in the acute sector or NHS Board head quarters (P<0.001); increasing duration of time since training (P<0.05) and belonging to the management or nursing & midwifery professional groups (P<0.05). Other factors such as the type and duration of training and respondents’ gender and job experience were not statistically associated with RCA involvement or leadership.

### Post-training involvement in RCA investigations

The majority of respondents reported that they had acquired no post-training involvement in any RCA investigations since being trained (127, 55.7%) and cited the following reasons: ‘*no opportunity*’ to do so (101, 86.6%); ‘*lack of support*’ (5, 3.9%); and ‘*inadequate training*’ (1, 0.8%).

Table 3 displays a numerical breakdown of respondents’ reported levels of RCA involvement. 101 respondents (44.3%) indicated that they had participated in an RCA investigation with 71 assuming a lead investigator role (70.3%) on one or more occasion. Of this group, around 41% had led one RCA investigation with almost 20% reporting a leadership role in five or more investigations since undergoing training.

**Table 3:** Respondents’ reported levels of RCA participation

**RCA Investigations**	**Respondents who led RCA investigations**	**Respondents who participated in RCA team investigations**	**Respondents who both led and participated in RCA investigations**
**(n)**	**n=71 (%)**	**n=74 (%)**	**n=101 (%)**
1	29 (40.8)	30 (40.5)	30 (29.7)
2	18 (25.4)	20 (27.0)	26 (25.7)
3	9 (12.7)	9 (12.2)	10 (9.9)
4	1 (1.4)	4 (5.4)	8 (7.9)
≥5	14 (19.7)	11 (14.9)	27 (26.7)

### RCA recommendations: implementation, benefits and barriers

A clear majority of respondents (83, 82%) indicated that the improvement recommendations made as part of their RCA investigations were generally implemented, or partly implemented. Table 4 outlines selected examples of the perceived benefits of participating in the RCA process and other comments about RCA practices that were reported by study participants.

**Table 4:** Comments by health care professional respondents on RCA practices

	
**Positive benefits**	
·	Working locally with teams and supporting staff during the process
·	Reflecting on the outcomes of the implementation of the recommendations and action plans, and observing the consequent improvements in patient safety and service delivery
·	Allowed you to step back and look objectively at the situation without being too close and avoid the wood for the trees scenario
·	Staff feeling there was a real attempt to look for improvements rather than blame
·	I believe it has brought reproductive medicine and obstetrics & gynaecology theatres together more as a team
·	Everyone involved could see the benefit of getting to the roots of the issue so that it wouldn’t happen again, or at least the risks of it happening were reduced
·	Reassuring staff that RCA is trying to identify ways of learning from mistakes…and not to punish.
·	Staff started to understand the importance of problem-solving and the breaking down of a blame culture
·	Team of people involved felt listened to and understood
·	Outcome reflection raised awareness and attitude change to how we can do things differently and improve the patient experience
·	Clarifying what was the actual cause of the problem. It is not always what seems obvious. And working out what can be done to prevent a recurrence.
·	Confidence that the situation was being examined in a systematic & comprehensive way
·	Getting the problem sorted in a positive manner and every one learning from it
**RCAs should be conducted by clinical staff only**	
·	Disagree, useful to have input from those removed from the situation.
·	Clinical input essential in clinical cases - but objective input to provide different perspective can be valuable in any investigation
·	I think assistance from a dedicated team might help, especially if you don’t have alot of experience in completing regularly.
·	It is of benefit to have a understanding of the area however not necessarily should an RCA be carried out by staff within department.
·	It depends very much on the incident reported who should be involved. There may be incidents such as case notes going missing which involved other groups of staff , not just those from a clinical background.
·	Yes but with help from other clinical staff who have undertaken an RCA
·	It should also involve staff out-with your own clinical area to allow a more balanced view i think.
·	needs a mix and definite somebody removed from situation
·	Should be conducted by colleagues within clinical background however fresh eyes can often help in some situations
·	No, the most appropriate person should lead. It's good on occasions to have someone independent
**Patients or relatives should be involved in RCA investigations**	
·	Yes I believe they should if they are willing
·	It would depend on the nature of the incident being investigated.
·	Possibly - would depend on the situation and training available.
·	No - will not necessarily be appropriate as emotions can develop and are not helpful.
·	It might be useful to have “specialist” patient/carer representatives involved - i.e. ones with some real experience of the patient/carer perspective plus RCA training, but who were not personally involved in the particular incident.
·	A patient liaison officer maybe more appropriate to avoid upset or distress to a patient or relatives
·	No, I think it would be difficult for others to share their opinion in discussion if they were present, but it may be appropriate at times to meet with relatives or patients before and/or after an RCA has been conducted

Table 5 outlines a range of commonly known barriers to conducting RCA investigations. In descending order, the top three barriers cited as ‘always’ or ‘sometimes’ encountered were lack of time (54, 54.6%), unwilling colleagues (33, 34%) and inter-professional differences (30, 31%). Just under 40% of respondents indicated that outside interference from internal or external sources was also a barrier to some degree during their RCA investigations.

**Table 5:** Respondents who participated in and led RCA investigations: reported barriers to RCA and extent to which recommendations were implemented (n=101)

**Survey item**	**Participants responding, n (%)**				
**Generally speaking, were your recommendations implemented? (n=98)**	**No**		**Partly**	**Yes**	**Unsure**
	1 (1.0)		42 (41.6)	41 (40.6)	14 (13.9)
**Did you encounter the following barriers?**	**Always**	**Sometimes**	**Unsure**	**Occasionally**	**Never**
Unwilling colleagues (n=97)	4 (4.1)	29 (29.9)	3 (3.1)	18 (18.6)	43 (44.3)
Unsupportive management (n=97)	1 (1.0)	12 (12.4)	2 (2.1)	11 (11.3)	71 (73.2)
Lack of resources (n=95)	6 (6.3)	14 (14.7)	11 (11.6)	33 (34.7)	31 (32.6)
Lack of time (n=99)	17 (17.2)	37 (37.4)	0 (0.0)	25 (25.3)	20 (20.2)
Interference from internal/external sources (n=96)	2 (2.1)	16 (16.7)	8 (8.3)	12 (12.5)	58 (60.4)
Difficulty with RCA teams (n=93	1 (1.1)	3 (3.2)	10 (10.8)	12 (12.9)	67 (72.0)
Lack of feedback and data (n=94	6 (6.4)	13 (13.8)	7 (7.4)	23 (24.5)	45 (47.9)
Inter-professional differences (n=97)	2 (2.1)	28 (28.9)	3 (3.1)	26 (26.8)	38 (39.2)
Mean for all barriers (%)	5.1%	19.8%	5.7%	20.8%	48.6%

### Attitudes to RCA training and the RCA process: group comparisons

#### RCA training

A clear majority of all respondents (178, 76.8%) indicated ‘yes’ or ‘partly’ to the question on whether they had sufficient understanding/confidence by the end of the training to conduct an RCA (Table 6). A smaller majority ticked ‘yes’ or ‘partly’ (49, 119, 51.3%) when reporting if their work practices regarding safety and reporting errors had changed since attending the RCA training course. Overall, respondents agreed that they were now: better trained in methods of dealing with incidents (76.5%); more able to improve work processes for the provision of safer clinical care; and of the belief that RCA training can contribute to the advancement of safety in healthcare. There was no clear statistical differences in responses to statements by both groups apart from one very obvious statement on whether the training provided respondents with the skills to be involved in or lead RCA in the workplace, with a greater proportion of those who had actually participated in RCA in agreement (P=0.008).

**Table 6:** Levels of agreement: attitudinal comparisons by respondent groups towards safety skills acquired and RCA impact

**Survey items**	**Group 1: Respondents who led or participated in RCA n=101 (%)**				**Group 2: Respondents who did not participate in or lead RCA n=127 (%)**				**All respondents n=228 (%)**				**Chi Square**		
	*Yes*	*Partly*	*No*	*Unsure*	*Yes*	*Partly*	*No*	*Unsure*	*Yes*	*Partly*	*No*	*Unsure*	*χ2*	*Df*	*P*
Did you have sufficient understanding/confidence by the end of the training to conduct a RCA?	56 (55.4)	24 (23.8)	12 (11.9)	6 (5.9)	59 (46.5)	39 (30.7)	15 (11.8)	8 (6.3)	115 (49.6)	63 (27.2)	27 (11.6)	14 (6.0)	1.874	3	0.60
Have your work practices regarding safety and reporting errors changed since you attended the RCA training?	53 (52.5)	8 (7.9)	33 (32.7)	2 (2.0)	47 (37.0)	11 (8.7)	59 (46.5)	3 (2.4)	100 (43.1)	19 (8.2)	92 (39.7)	5 (2.2)	5.786	3	0.122
	*Agree*	*Unsure*	*Disagree*		*Agree*	*Unsure*	*Disagree*		*Agree*	*Unsure*	*Disagree*		*χ2*	*Df*	*P*
Have you been able to apply the knowledge gained from your RCA training to your workplace?	38 (37.6)	3 (3.0)	59 (58.4)		46 (36.2)	8 (6.3)	73 (57.5)		84 (36.8)	11 (4.8)	132 (57.9)		1.327	2	0.515
Since undertaking RCA training do you think you are better trained in methods of dealing with incidents?	84 (83.2)	14 (13.9)	3 (3.0)		90 (70.9)	33 (26.0)	2 (1.6)		174 (76.3)	47 (20.6)	5 (2.2)		5.602	2	0.061
Since undertaking RCA training can you improve work processes for provision of safe clinical care?	78 (77.2)	20 (19.8)	2 (2.0)		77 (60.6)	43 (33.9)	4 (3.1)		155 (68.0)	63 (27.6)	6 (2.6)		6.574	2	0.037
Over the long term, will RCA training contribute to the advancement of safety in healthcare?	85 (84.2)	16 (15.8)	0 (0.0)		97 (76.4)	26 (20.5)	1 (0.8)		182 (79.8)	42 (18.4)	1 (0.8)		1.84	2	0.399
In general, did the RCA training provide you with the skills to be involved in or lead a RCA?	78 (77.2)	16 (15.8)	7 (6.9)		71 (55.9)	39 (30.7)	13 (10.2)		149 (65.4)	55 (24.1)	20 (8.8)		9.68	2	0.008

#### RCA process

Respondents with post-training experience of RCA investigations were more likely to agree that the benefits associated with training are worth the investment (73.2% v (65.4%) and that conducting RCA was a good use of staff time (86.2% v 77.6%) than those with no post-training experience, although overall agreement with the statements was high (Table 7). Similar differences in levels of agreement between both groups were noted when asked if a follow-up session after conducting RCA would be beneficial (65.3% v 39.4%) and if receiving confidential peer feedback on the RCA report would benefit their learning (83.2% v 37.0%), although significant minorities were unsure in both instances. In terms of who should be involved in RCA investigations, respondents had divided views on whether these should be conducted by clinical staff only, with well over half disagreeing or indicating that they were unsure about this (116/179, 64.8%). A small majority of respondents (101/178, 56.7%) answered ‘agree’ or ‘maybe’ to the statements on whether patients or relatives should be part of the RCA team (see Table 4, for related comments).

**Table 7:** Levels of agreement: attitudinal comparisons by respondent groups towards value of RCA process and need for further education

**Survey items**	**Group 1: Respondents who led or participated in RCA n=101 (%)**			**Group 2: Respondents who did not participate or lead RCA n=127 (%)**			**All respondents n=228 (%)**			**Chi Square**		
	*Agree*	*Unsure*	*Disagree*	*Agree*	*Unsure*	*Disagree*	*Agree*	*Unsure*	*Disagree*	*χ2*	*Df*	*P*
Considering the health systems investment in RCA training, are the benefits you see worth the investment?	74 (73.3)	21 (20.8)	5 (5.0)	75 (59.1)	45 (35.4)	4 (3.1)	149 (65.4)	66 (28.9)	9 (4.0)	6.347	2	0.042
Undertaking a RCA is a time-consuming business. Is it good use of staff time and resources?	87 (86.1)	10 (9.9)	4 (4.0)	90 (70.9)	28 (22.0)	5 (3.9)	177 (77.6)	38 (16.7)	9 (3.9)	6.591	2	0.037
	*Yes*	*No*	*Unsure*	*Yes*	*No*	*Unsure*	*Yes*	*No*	*Unsure*	*χ2*	*Df*	*P*
Do you think a follow-up training session after you had actually conducted a RCA would be beneficial?	66 (65.3)	8 (7.9)	23 (22.8)	50 (39.4)	3 (2.4)	19 (50.0)	116 (50.0)	11 (4.7)	42 (18.1)	1.188	2	0.552
Would receiving confidential peer-feedback on your final RCA report be beneficial as part of your learning?	84 (83.2)	1 (1.0)	16 (15.8)	47 (37.0)	1 (0.8)	15 (11.8)	131 (56.5)	2 (0.9)	31 (13.4)	1.773	2	0.412

### Comparison with selected study findings of Braithwaite et al. (2008)

Similar to the previous Australian study [18] we found no differences in the professional characteristics of those who had conducted a post-training RCA and those who had not, while those staff in both studies with RCA experience had undertaken multiple investigations. However, we found that a greater proportion of respondents in our study had led a post-training RCA investigation compared with the earlier Australian study [(76/101, 75.2% v 133/252, 52.8%, diff=22.4%, 95% CI 11.4 to 32.1%, P<0.001)]. We also noted that our respondents cited (‘always’ and ‘sometimes’) the following barriers less frequently than their Australian counterparts: ‘lack of time’ [(54/99, 54.6% v 189/252, 75.0%, diff=20.5%, 95% CI 9.4 to 31.4%, P<0.001)]; ‘difficulty within RCA teams’ [(4/93, 4.3% v 86/252, 34.2%, diff=29.8%, 95% CI 21.5 to 36.4%, P<0.001)]; ‘unwilling colleagues’ [(33/97, 34.0% v 112/252, 44.5%, diff=10.4%, 95% CI −1.1 to 21.1%, P=0.077)]; and ‘lack of feedback and data’ [(19/94, 20.2% v 96/251, 38.3%, diff=8.1%, 95% CI 7.2 to 27.3%, P=0.002)]. A further significant difference was highlighted in terms of RCA improvement recommendations with a greater proportion of Scottish respondents reporting that these were implemented or partly implemented [(83/98, 84.5% v 175/252, 69.4%, diff=15.1%, 95% CI 5.3 to 23.6%, P=0.004)].

## Discussion

The main findings provide a measure of the extent to which healthcare staff trained in RCA actually participated in subsequent investigations in the workplace. It is clear in this study that a majority has yet to do so with the greatest proportion of trained but inexperienced staff being based out-with the acute care sector. This is a concern given the resources committed to RCA training and the implications in terms of lost opportunities to improve patient safety and organizational learning, as well as the potentially discouraging impact on staff morale and attitudes. Previous research has reported concerns from RCA-trained staff that they may lack ‘*personal control*’ over participation in subsequent incident investigations which is likely, for many, to be a decision for local managers in their organisations and heavily dependent upon workload priorities [16-18,20].

The ‘*failure of work schedules*’ to provide staff with protected time for RCA investigations was cited as *the* major difficulty by Braithwaite et al. [18]. Edmonson (2004) suggests that leadership has a critical role in this regard in developing an environment of ‘*psychological safety*’ to encourage a greater willingness for transparency, questioning and sharing of concerns, and also in ‘*supporting and empowering*’ team learning across the organisation [21]. Nicoloni et al. (2011) also suggest that leaders need to openly endorse RCA (or a variant) as an improvement method and the staff who have been trained to implement it [8]. Without this type of approach then it is possible that local leaders will continue to forfeit opportunities to learn from RCA by occasionally or even frequently assigning a low priority to investigations or blocking participation in the process by trained staff.

Most respondents with RCA experience report multiple exposures to incident investigations. Similar to previous research [9-11,16-19,21,22], a clear majority also report that their recommendations to make care safer are at least partly or fully implemented in their organisations, although determining how effective these potential improvements actually were was not a study aim but nonetheless should be a subject of further research given the limited evidence.

It is possible, perhaps even likely, that RCA investigators will be subject to some form of ‘criticism’ or ‘conflict’ from ‘powerful’ individuals or groups, or those with vested interests, within a healthcare organisation [22]. It should also be remembered that in essence RCA involves healthcare professionals ‘*not just scrutinizing each other but scrutinizing each others’ errors*’. Many respondents encountered a range of organizational barriers to conducting RCA investigations similar to those reported previously [18]. A few significant differences were apparent, however, with respondents in our study more likely to adopt a post-training RCA leadership role and also report less difficulty with some of the organizational barriers outlined. One interpretation is that the differences indirectly hint at a slightly more positive organizational safety culture being reported in our study, which is possible given the large-scale national initiatives to improve patient safety in the Scottish health service over recent years [15]. However a more likely explanation is that the Australian study [18] is more than five years older and so associated cultural factors such as the prevailing attitudes and behaviours towards RCA investigations and patient safety in general may have improved over time to match those in our study findings – not that the results reported here offer some type of benchmark.

Similar to other studies, our RCA-experienced respondents were generally positive about the training they received and the cost-benefits of this investigation technique in terms of the ‘*advancement of safety in healthcare*’ and being a ‘good *use of staff time and resources*’ [16,18,20]. Respondents displayed mixed views - supported by seemingly logical arguments on both sides - on whether non-clinical colleagues and patients should have a role in incident investigations. However, many risk managers are non-clinical and this does not seem to have been a barrier to them leading on RCA training or advising on, or participating in, related investigations. The involvement of patients and relatives in these investigations is strongly encouraged in national policy [12-15], but the reality is that this appears to be a rarity perhaps because of the many sensitivities and difficulties outlined by our respondents, even if many were positive about the prospect.

Most respondents had a sufficient ‘understanding/confidence’ in conducting RCA and indicated that their work practices and reporting of errors have changed since being trained. A follow-up training session and the use of confidential peer feedback on RCA reports were viewed as potentially beneficial educational interventions, suggesting that many respondents may have a level of insight into the need for further learning around the often complex and problematic issues involved in applying the technique. The inconsistent quality of RCA attempts and the need for additional post-training support has been noted previously [17,19]. The study by Wallace et al. (2009) reports high levels of satisfaction with RCA training, but low levels of correct responses when study participants were subsequently tested on their acquired knowledge of RCA using pre-designed vignettes [16].

Overall the evidence demonstrates that the standard of incident analysis and report writing is frequently variable [7,16,17,19]. Given that the written report is a key proxy for the quality of the investigation undertaken then it is likely that some type of educational feedback intervention is necessary. Offering developmental support and mentorship, particularly to guide less experienced staff confronting and dealing with some of the aforementioned barriers to RCA, and when writing comprehensive unambiguous reports that offer realistic recommendations for improvement (either during training or after training or both), arguably makes sense in closing this educational gap. Taken together the combined evidence from this and other studies cited may point to an organisational learning need for continuous development and feedback for RCA-trained staff, or at least in the short-term [7,16,18,20].

The study findings are not generalisable beyond the RCA training practices in this single health authority. But given the degree of congruence with Braithwaite et al. [18] and specific findings in previous research [9-11,16,17,19,21,22], it is possible that similar issues would be uncovered in other regions and countries with comparable training arrangements, particularly with regard to the significant proportion of trained staff who do not gain any post-training investigation experience. A key consideration, therefore, will be the cost-benefits involved in taking healthcare staff out of frontline clinical duties to provide them with RCA training and then failing to utilize or support them in the post-training phase.

One potential option is to better select staff for more intensive RCA training, while training less staff numbers so that organisations develop a strong core grouping with the requisite experience, expertise and leadership skills - augmented by the provision of continuous developmental support. Potentially this offers a number of advantages over current arrangements. A better trained and dedicated RCA staff group which is afforded greater opportunities to gain experience may retain and strengthen their analytical knowledge and skills and also benefit from shared peer-to-peer learning – leading to more meaningful and effective incident investigations. In developing this ‘expert community of practice’, these individuals (or as a group) may also become better equipped to highlight and challenge existing institutional barriers to engaging in and learning from incident investigation and start to make progress in developing a more positive safety culture. However, this will require organisations – and, perhaps more specifically, local healthcare leaders - to give greater priority to investigations and provide some element of protected time for these staff to continue to develop related experience and expertise when necessary. In some cases this may require a paradigm shift in local middle management and executive level attitudes and behaviours towards improving patient safety that goes beyond purely rhetorical endorsement of this concept as *the* single most important healthcare priority. A recent high profile media exposure of inconsistencies around serious patient safety incident investigation practices across NHSiS may have some impact in this regard [23].

### Strengths and limitations

Our survey generated a moderate response rate although respondent numbers were still significantly large enough for useful statistical inferences and adding to our knowledge and understanding in this topic area. A number of limitations are associated with this type of descriptive cross sectional survey. It is likely that a proportion of non-respondents will have changed posts and therefore email addresses since RCA training and so could not be tracked using the online survey system. There may have been response bias as we were unable to compare and explore the characteristics of responders and non-responders as well as recall bias given the time lag between training and completing the questionnaire experienced by some. Also, self-report data may not be fully reliable as there is no means of independent verification. Caution should therefore be exercised when extrapolating these findings for more general purposes.

## Conclusion

The study quantified some important problems within a single NHS board’s RCA training programme which will be of wider interest in Scotland and internationally. This adds to our knowledge and understanding of the need to improve the effectiveness of related training and frontline practices in healthcare settings. There is an assumption that organizations can train staff in RCA and learn from associated outcomes as if it is a linear, “*rational, robust and rigorous process*” [8,22]. However, healthcare authorities may wish to look more critically at the system and cultural complexities which impact RCA investigations; the professional groupings and numbers of staff whom they select to attend training; and how these programmes are delivered and supported educationally in the longer term to maximize cost-benefits, organizational learning and safer patient care. A deeper understanding of the socio-cultural issues at play is also necessary, but this will require a policy commitment to resource more in-depth social research and evaluation, particularly if developing, testing and implementing new training paradigms.

## Competing interests

The authors declare there are no competing financial or non-financial interests.

## Authors’ contributions

PB conceived the study idea, acquired funding, co-led the study design, data collection, analysis and interpretation, and drafted the initial manuscript. JS co-led the study design and data collection, and contributed to the content and critical review of the manuscript. CdW led on the statistical analysis and interpretation, and contributed to the content and critical review of the manuscript. All authors read and approved the final manuscript.

## Pre-publication history

The pre-publication history for this paper can be accessed here:

http://www.biomedcentral.com/1472-6963/13/50/prepub
